# Prevalence and Antibiotic Resistance Patterns of* Campylobacter* spp. Isolated from Broiler Chickens in the North of Tunisia

**DOI:** 10.1155/2018/7943786

**Published:** 2018-12-23

**Authors:** Manel Gharbi, Awatef Béjaoui, Cherif Ben Hamda, Ahlem Jouini, Kais Ghedira, Chahrazed Zrelli, Safa Hamrouni, Chedia Aouadhi, Ghaith Bessoussa, Abdeljelil Ghram, Abderrazek Maaroufi

**Affiliations:** ^1^Laboratory of Epidemiology and Veterinary Microbiology, Group of Bacteriology and Biotechnology Development, Institut Pasteur de Tunis, University of Tunis El Manar (UTM), BP 74, 13 Place Pasteur, Belvédère, 1002 Tunis, Tunisia; ^2^Laboratory of Bioinformatics, Biomathematics and Biostatistics, Institut Pasteur de Tunis, University of Tunis El Manar (UTM), Tunisia; ^3^Faculty of Science of Bizerte, University of Carthage, 7021 Jarzouna, Tunisia

## Abstract

The aim of the current study is to assess the prevalence of* Campylobacter* infection in broiler chickens, raised in intensive production conditions, and to evaluate the antimicrobial susceptibility of recovered* Campylobacter* isolates. A total of 590 cloacal swab samples were taken from 13 broiler chicken flocks in the North East of Tunisia. All samples were tested for the presence of thermophilic* Campylobacter* by culture and PCR, targeting the* mapA *and* ceuE* genes, respectively. Susceptibility to antimicrobial drugs was tested against 8 antibiotics. Prevalence of* Campylobacter* infection, relationship with geographic origins and seasons, antimicrobial resistance rates and patterns were analyzed. Total prevalence of* Campylobacter* infection in broiler flocks was in the range of 22.4%, with a predominance of* C. jejuni* (68.9%), followed by* C. coli *(31.1%). Positive association was highlighted between the infection level and the season (*P* < 0.001), but no link was emphasized considering the geographic origin. Antimicrobial susceptibility testing revealed very high resistance rates detected against macrolide, tetracycline, quinolones, and chloramphenicol, ranging from 88.6% to 100%. Lower resistance prevalence was noticed for *β*-lactams (47% and 61.4%) and gentamicin (12.9%). 17 R-type patterns were observed, and a common pattern was found in 30.3% of isolates. This study provides updates and novel data on the prevalence and the AMR of broiler campylobacters in Tunisia, revealing the occurrence of high resistance to several antibiotics and emphasizing the requirement of better surveillance and careful regulation of antimicrobials use.

## 1. Introduction

Campylobacteriosis is a major food-borne zoonosis with global distribution [1]. In last decade, the number of human campylobacteriosis cases has increased in both industrialized and developed countries, with 96 million cases of gastroenteritis and 21 thousand deaths per year, worldwide [2–4]. In fact, diarrheal illness is particularly important in developing countries, where infection with these pathogens in children under the age of two years may lead to death [5]. The majority of human infections are caused by* C. jejuni* (80-85%), whereas most of the remaining cases are attributed to* C. coli* [6]. Even though epidemiological data from Africa, Asia, and Middle East are still incomplete, available data indicate that* Campylobacter* infection is endemic in these regions [2]. Poultry is the main reservoir of these pathogens and harbors them without clinical manifestations. Transmission of* Campylobacter* to humans occurs mainly through consumption of contaminated raw/undercooked poultry products, or through close contact with infected animals [7, 8]. A small proportion of campylobacteriosis cases may be attributed to other animals or environmental sources [9]. In the EU, it has been estimated that 50 to 80% of human cases of campylobacteriosis may be attributed to the chicken reservoir, whereas the handling, preparation, and consumption of contaminated broiler meat may account for 20 to 30% of cases [10]. The majority of human* Campylobacter* infections results in a self-limiting gastroenteritis illness and does not require specific treatment. However, severe, prolonged or systemic infections, in immunocompromised, vulnerable population and children may require antimicrobial therapy. The macrolides (erythromycin) are usually the first-line drugs, whereas fluoroquinolones and, to a lesser extent, tetracycline represent alternative options [11]. Not long ago,* Campylobacter* has developed resistance to several antimicrobial agents, including macrolides, tetracycline, and quinolones [12]. Multidrug-resistant* Campylobacter* strains have been frequently isolated, and this could complicate the treatment of human infection [13]. In addition to the campylobacteriosis morbidity and the risk to develop long term sequelae (e.g., Guillian Barre' syndrome (GBS) and reactive arthritis), the development of resistance to antimicrobial drugs by* Campylobacter* strains constitutes an important concern. Thus, the development of effective mitigation strategies for* Campylobacter* reduction in broiler, as well as the successful use of antimicrobial treatment, requires a good understanding of the epidemiological status of* Campylobacter* infection in flocks, in order to decrease the prevalence of this infection and likely antimicrobial resistance.

The aim of the present study is to assess thermophilic* Campylobacter* prevalence in broiler flocks in North East of Tunisia and to determine antimicrobial resistance rates and patterns of recovered* Campylobacter* strains.

## 2. Material and Methods

### 2.1. Sampling

Samples were collected from 13 broiler farms, between December 2016 and May 2018, located in three governorates including Ariana, Ben Arous, and Nabeul (in the North East of Tunisia). These areas ensure 29% of national broiler production [14]. From each farm, 30 to 50 cloacal swabs were taken from randomly selected birds. All farms have similar breeding and biosecurity/biosafety protocols.

### 2.2. Isolation of Campylobacter

Overall, 590 cloacal swabs were taken from chickens. Samples were inoculated into Bolton Broth (Oxoid, UK) and incubated, for enrichment, at 42°C for 48 h in a microaerobic environment (5% O_2_, 10% CO_2_ and 85% N_2_), created by GENbox generators (BioMerieux, France). A loopful of each enriched sample was streaked on Karmali plates (SIGMA-ALDRICH) and incubated under the same conditions, as described above. From each sample, a total of ten swarming, opaque, white to grey colonies suspected of being campylobacters were subcultured on Karmali agar. Colonies comprising curved or spiral motile rods, when observed by light microscopy, were examined for oxidase/catalase activities and Gram stained. Thereafter, a maximum of three microscopically confirmed* Campylobacter* isolates per sample were subjected to PCR analyses for genus confirmation and species identification.

Confirmed* Campylobacter* isolates were stored in brain heart infusion broth (Bio-Rad) with 25% glycerol at −80°C for further analysis. Only one isolate per positive samples was used for further analyses.

### 2.3. Molecular Identification

For PCR analysis, template DNAs were prepared by boiling method. Presumptive* Campylobacter* spp. colonies were selected from Karmali agar and cultured in Bolton Broth for 24h, in the same conditions as described above. A volume of 100 *μ*l of culture was centrifuged at 13,000 ×* g* for 10 min and the supernatant was carefully discarded. The bacterial pellet was suspended in 500 *μ*l of TE buffer [10 mM Tris-HCl, 1 mM EDTA (pH 8.0)] and heated by immersion in a boiling water bath for 10 min. The samples were cooled immediately on ice for 5 min and centrifuged at 13000 × g for 5 min. The supernatant was recovered and stored at -20°C, until analysis.

Genus confirmation of presumed* Campylobacter* isolates was performed by PCR amplification of a specific fragment of 16S* rRNA* gene, using previously described primers [15]. The isolates were confirmed as* C. jejuni* or* C. coli* by PCR assays based on* mapA* and* CeuE* genes amplification, respectively [16, 17]. The sequence and the origin of the three sets of used primers are indicated in Table 1.

All PCR reactions contained 2.5 *μ*l DNA template, 0.2 *μ*M of each primer (Carthagenomics Advanced Technologies, Tunisia), 0.2 mM of dNTP (PROMEGA), 1X Dream Taq DNA polymerase buffer, and 1.0 U of Dream Taq DNA polymerase (Thermo Scientific), in a final reaction volume of 25 *μ*l.

The PCR protocol for genus identification was as follows: 5 minutes at 95°C, 35 cycles consisting of 1 min at 95°C, 1 min at 55°C, 1 min at 72°C, and a final extension step of 10 minutes at 72°C. The same protocol was used for species identification, except for annealing temperature, which was at 59°C. All DNA amplification reactions were carried out in a T100 thermal cycler (BIO-RAD).

For the visualization of PCR products, 10 *μ*l was subjected to electrophoresis on agarose gel containing ethidium bromide, and bands were visualized with UV light.

*C. jejuni* (ATCC 33291) and* C. coli* (CCUG 11283-T) were included in all tests as positive controls.

### 2.4. Antimicrobial Susceptibility Testing

*Campylobacter* isolates were tested for susceptibility to antimicrobial drugs by disk diffusion method, as recommended by the European Committee on Antimicrobial Susceptibility Testing (EUCAST) [18]. Bacterial suspension of 0.5 McFerland was prepared from 24h culture and inoculated on Mueller-Hinton agar (Becton Dickinson) plates supplemented with 5% defibrinated sheep blood. All isolates were tested with the following antibiotics: ampicillin (**AM**: 10 *μ*g), amoxicillin/clavulanic acid (**AMC**: 20/10 *μ*g), gentamicin (**GEN**: 10 *μ*g), erythromicin (**ERI**: 15 UI), nalidixic acid (**NA**: 30 *μ*g), ciprofloxacin (**CIP**: 5 *μ*g), tetracycline (**Tet**: 30 *μ*g), and chloramphinicol (**CHL**: 30 *μ*g). Plates were incubated at 37°C for 24h in microaerophilic atmosphere.* C. jejuni* (ATCC 33291) was used as quality control organism.

Inhibition zones were measured and interpreted as recommended by EUCAST's criteria [18]. Isolates exhibiting phenotypic resistance to three or more antimicrobial classes were regarded as multidrug resistant.

### 2.5. Data Analysis

All the data collected within the present study were analyzed using R software, a language and environment for statistical computing [19]. The antimicrobial resistance analyses were performed by means of a Chi-square statistic (P < 0.05) [20]. This test is a nonparametric tool designed to compare frequency counts between two groups of different sample sizes. Comparison between isolate prevalence across season in three Tunisian governorates was carried out by means of a one-way analysis of variance (ANOVA) [21], followed by a TukeyHSD test (Tukey Honest Significant Differences). This later is a post hoc test based on the studentized range distribution (q Statistics) [22]. The selection criterion for significantly prevalence variance was a stringent p value of 0.001 or less.

## 3. Results

### 3.1. Thermotolerant Campylobacter spp. Prevalence

The present study revealed that the prevalence of* Campylobacter* spp. was of 22.4% (132/590). A total of 132 isolates was recovered from cloacal swabs, from 10 out of 13 investigated flocks.

When looking at the distribution of* Campylobacter* spp.,* C. jejuni* was the prominent isolated species (68.9%), followed by* C. coli* (31.1%). Five samples collected from the same farm were found containing both* C. jejuni* and* C. coli* (0.8%).

The prevalence of infected flocks ranged from 6.1% to 56%, and the prevalence of infection per governorate ranged from 11.1% to 54%. Regarding, the distribution of both thermophilic species, no significant difference (*P* < 0.001) was noticed regarding flocks or governorates.

**Figure 1:**
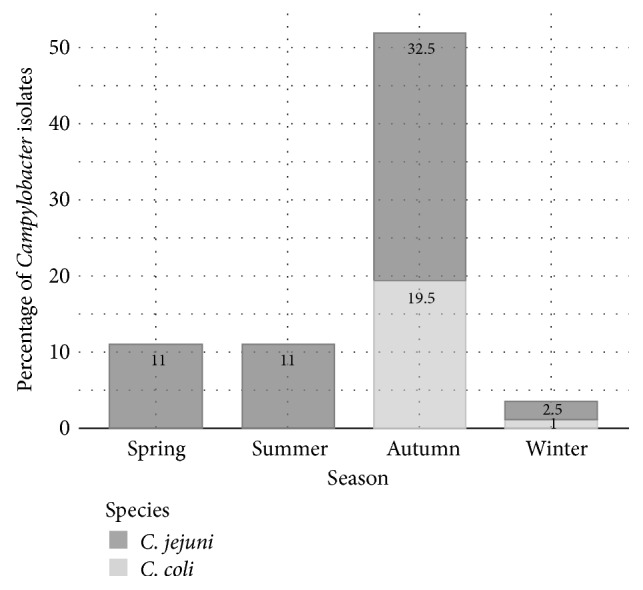


On the other hand, we have shown that the highest prevalence of* Campylobacter* spp. infection was found in autumn season (52%) and the lowest during the winter season (3.5%). In spring and summer, we have obtained the same rate of infection (11%) (Figure 1). The difference between seasonal prevalence was statistically significant (*P* < 0.001).

### 3.2. Antibiotic Susceptibility of Isolated Strains

Results of antimicrobial susceptibility of both* C. jejuni* and* C. coli* isolates against the eight tested antimicrobial agents are shown in Table 2.

All isolates (100%) were resistant to tetracycline and erythromicin, and a very high resistance was observed against ciprofloxacin (99.2%) and chloramphenicol (88.6%). To a lesser extent, resistance rates to *β*-lactams were about 61.4% to ampicillin and 47.0% to amoxicillin/acid clavulanic. Similar resistance levels were also observed for the nalidixic acid (46.2%). The resistance rates between* C. jejuni* and* C. coli* isolates against AM (73.6% and 34.1%) and NA (57.1% and 22%, respectively) differ significantly (*P* < 0.05). No significant difference was observed between both species for the other AMD.

Multidrug resistance to three antimicrobial classes was detected in all* Campylobacter* isolates and frequency of resistance profile including 4, 5, and 6 AMC was as follows; 31.8%, 53.8%, and 14.4%, respectively.

When looking at the AMR patterns, 17 R-types were found for all* Campylobacter* isolates (Table 3), with the combination “CIP, ERI, TET, and CHL” as the most common profile (30.3%). A significant higher percentage of* C. coli* strains (63.4%) belonged to this group, compared with* C. jejuni* isolates (15.4%). The next most frequent pattern was the combined resistance to “AM, AMC, NA, CIP, ERI, TET, and CHL”, detected in 21.2% of isolates. The remaining patterns comprised less than 10% of isolates. For* C. coli,* only four patterns composed of 4, 5 (2 patterns), and 6 antimicrobial classes were detected. One of these patterns corresponding to “AM, AMC, CIP, ERI, TET and CHL” combination seems to be specific to* C. coli* isolates (n=5; 3.8%).

## 4. Discussion

Poultry is the main reservoir of* Campylobacter* and its meat consumption represents a major cause of human campylobacteriosis. The presence of such pathogens in poultry is of great concern for human health, and their control in broiler flocks has a great impact on public health.

In Tunisia, as in many developing countries, there is limited information regarding* Campylobacter* status in conventional broiler flocks. Our findings demonstrated that the prevalence of* Campylobacter* in broiler flocks was about 22.4%. This prevalence can be considered as moderate compared to those reported by other studies in different countries. Indeed, studies in Brazil, Costa Rica, and Sri-Lanka showed that 100.0%, 80.0%, and 63.8%, of flocks were, respectively,* Campylobacter* positive [23–25]. The European baseline study on* Campylobacter* in broilers indicated that the EU prevalence was 71.2%, ranging from 2% in Estonia to a maximum of 100% in Luxembourg [10]. The prevalence assessed in the present study is higher than prevalence shown in the European Nordic countries, such as Sweden (13%), Finland (3.9%), and Denmark (10.3%), but lower than prevalence reported in Spain (88%), Portugal (82%), and France (76.1%).

Regarding* Campylobacter *species distribution,* C. jejuni* was the most common species (68.9%) found, followed by* C. coli* (31.1%). These results are similar to other investigations reporting* C. jejuni* and* C. coli* as the most frequently isolated species in poultry [26, 27] and their distribution is comparable to our results (Ireland: 68.9% and 32.4%; Austria 65.1% and 33.3% for* C. jejuni* and* C. coli*, respectively) [10].

The prevalence of* Campylobacter* in positive flocks at the governorate level ranged from 11.1% to 54%, and those within flocks varied from 6.1% to 56%. No significant difference was found related to region distribution, since the three sampled governorates belong to the same geographic region and have similar climatic conditions. Regarding the seasonal distribution, the highest positive percentage was found in flocks sampled in autumn, against a lower prevalence in winter (3.5%). Thus, we can admit that high prevalence of* Campylobacter* is related to the season rather than to other factors. In fact, in autumn the weather in Tunisia is warm and humid and the highest Heat Index (HI) values are noted in September and October [28]. These weather conditions combined with the type of poultry rising (in house) seem to be favorable for the survival of campylobacters in the environment and to enhance their spread in flocks [25]. This positive association is in agreement with data reported by the literature and supported by the seasonality feature of this disease in human, with a peak incidence always observed during warm and humid seasons [29].

Furthermore, the moderate prevalence of* Campylobacter* observed in our study is probably associated with the management practices and the biosecurity measures, implemented in the country to control bacterial infections (particularly* Salmonella*) in broiler chain production. In fact, all farms integrated in this study are composed of conventional broiler chicken house for intensive production, with similar management practices.

Moreover, it was shown in several studies that the risk of* Campylobacter* spread in a flock is directly related to the age of birds, and it increases with the presence of older flocks [30]. However, in our study we have included only broiler farms with birds aged no more than 6 weeks, which might explain, in part, this moderate prevalence.

Additionally, the susceptibility of recovered* Campylobacter* isolates against different antimicrobial drugs was investigated. Our results highlighted that* C. coli* and* C. jejuni* present high resistance rates to erythromycin, tetracycline, ciprofloxacin, and chloramphenicol and lower rates to nalidixic acid, ampicillin, amoxicillin/acid clavulanic, and gentamicin.

These high resistance rates for ERI (100%), TET (100%), and CIP (99.2%) are comparable to those reported in Italy [25, 31] and can be explained by the common use of the same antimicrobials as first-line drugs in broiler farms to combat bacterial infections. In fact, studies have shown clear positive association between the use of fluoroquinolones in poultry production and a resistance increase of* Campylobacter* isolates from chicken and human origins [32]. Whereas in countries not permitting the use of fluoroquinolones in poultry production, such as Australia and the Nordic European countries, few resistant* Campylobacter* isolates were found in chickens and humans [33]. Taken together, the high levels of antibiotic resistance are obviously related to the irrational and uncontrolled use of antimicrobial drugs.

On the other hand, the low antimicrobial resistance rate to gentamicin (12.9%) might be due to the infrequent usage of this antibiotic in poultry production because of its high cost. At a second level, the resistance to *β*-lactams (61.4% and 47.0%) can be considered as moderate and these results could reflect national initiatives to fight the misuse of such antibiotics. In fact, because of the high resistance levels to these antimicrobial drugs during the last few years in humans, their administration was considerably reduced. The decreased use of these AMD could likely influence positively their efficacy when used again.

When looking at the resistance rates within species, we have noted a significant difference to the AM and the NA between* C. jejuni* and* C. coli*; nevertheless, the relationship between species and resistance pattern is not yet clear.

All* Campylobacter* isolates were identified as MDR, and combined patterns comprising more than three AMC were found in all isolates. Such alarming resistance rates were reported in several countries, like Algeria (100%) [34], Italy (100%) [35], China (86%), and Pakistan (90.4%) [36].

In addition to our findings, multiple studies have shown that AMR is a significant problem in Tunisia [37], and this worrisome phenomenon is worsen by the lack of national antimicrobial surveillance systems. Nevertheless, national health authorities and experts (clinicians, epidemiologists, microbiologists…) are fully aware of the magnitude of this issue. Thus several efforts based mainly on an education strategy and on the restriction of the availability of selected antimicrobial agents, to streamline their use in husbandry and in public health, have been supported to be implemented in the country.

## 5. Conclusions

The current study provides data on the occurrence of* Campylobacter* infection in broiler flocks and the antibiotic resistance, in Tunisia. The high resistance rates and the emergence of multidrug resistant strains to several antimicrobial classes are alarming. Therefore, the implementation of specific control procedures for AMR surveillance and the adoption of a One Health approach are becoming mandatory, to prevent the emergence and the spread of resistant* Campylobacter* strains.

## Figures and Tables

**Table 1:** Primers sequence used for *Campylobacter* spp. identification and expected amplicon sizes.

**Species**	**Gene**	**Sequence (5**′**/3**′**)**	**Amplicon (bp)**	**References**
*C*. spp	*rRNA*	GGATGACACTTTTCGGAGC- CATTGTAGCACGTGTGTC	816	Linton et al. 1996

*C. jejuni*	*mapA*	CTATTTTATTTTTGAGTGCTTGTG GCTTTATTTGCCATTTGTTTTATTA	589	Stucki et al. 1995

*C. coli*	*ceuE*	AATTGAAAATTGCTCCAACTATG TGATTTTATTATTATTTGTAGCAGCG	462	Gonzalez et al. 1997

**Table 2:** Percentage of resistant *Campylobacter* isolates.

**Antimicrobial**	***C. jejuni*** % (n=91)	***C. coli*** % (n=41)	**Total** % (n=132)
Ampicillin	73.6^∗^ (67)	34.1 (14)	61.4 (81)
Amoxicillin/acid clavulanic	52.7 (48)	34.1 (14)	47.0 (62)
Ciprofloxacin	98.9 (90)	100 (41)	99.2 (131)
Nalidixic Acid	57.1^∗^ (52)	22.0 (9)	46.2 (61)
Erythromycin	100 (91)	100 (41)	100.0 (132)
Tetracycline	100 (91)	100(41)	100.0 (132)
Chloramphenicol	83.5 (76)	100 (41)	88.6 (117)
Gentamycin	14.3 (13)	9.8 (4)	12.9 (17)

^*∗*^Significantly higher resistance (P < 0.05) of C.* jejuni* compared with *C. coli*.

**Table 3:** Multidrug resistance profiles of *Campylobacter jejuni* and *Campylobacter coli*.

**Antimicrobial resistance pattern**	**No. of AMC** ^∗^	***C. jejuni*** **(n=91) **%		***C. coli*** **(n=41) **%	
		No	%	No	%
CIP ERI TET CHL	**4**	14	15.4%	26	63.4%
AM AMC NAL CIP ERI TET	**4**	2	2.2%	0	0
AMC CIP ERI TET CHL	**5**	2	2.2%	0	0
AM NAL CIP ERI TET	**5**	7	7.7%	0	0
AM CIP ERI TET CHL	**5**	12	13.2%	0	0
AM AMC CIP ERI TET	**5**	3	3.3%	0	0
AMC NAL CIP ERI TET CHL	**5**	6	6.6%	0	0
AM NAL CIP ERI TET GEN	**5**	2	2.2%	0	0
AM AMC CIP ERI TET CHL	**5**	0	0	5	12.2%
AM AMC NAL ERI TET CHL	**5**	2	2.2%	0	0
AM NAL CIP ERI TET CHL	**5**	4	4.4%	0	0
AM AMC NAL CIP ERI TET CHL	**5**	23	25.3%	5	12.2%
AM CIP ERI TET CHL GEN	**6**	3	3.3%	0	0
AMC CIP ERI TET CHL GEN	**6**	2	2.2%	0	0
AM AMC CIP ERI TET CHL GEN	**6**	2	2.2%	0	0
AMC NAL CIP ERI TET CHL GEN	**6**	2	2.2%	0	0
AM AMC NAL CIP ERI TET CHL GEN	**6**	5	5.5%	5	12.2%

^*∗*^AMC: antimicrobial class.

## Data Availability

All data generated or analyzed in this study are included in this published article. Nevertheless, we declare that detailed data that support the findings of this study can be available upon request from the corresponding author.
